# Nodular Fasciitis of the Breast Mimicking Breast Cancer

**DOI:** 10.1155/2014/747951

**Published:** 2014-05-20

**Authors:** Shinya Yamamoto, Takashi Chishima, Shouko Adachi

**Affiliations:** Department of Breast Surgery, Yokohama Rosai Hospital, 3211 Kozukue-cho, Kouhoku-ku, Yokohama 222-0036, Japan

## Abstract

Nodular fasciitis is a benign proliferative lesion that is usually found in the soft tissue of the upper extremity and trunk in young to middle-aged persons. It has rarely been described in the breast. A 35-year-old woman had noticed a mass in her left breast. It was elastic-hard, 13 mm in size, and located mainly in the upper inner quadrant of the left breast. Mammography did not detect the mass. Ultrasonography revealed a hypoechoic lesion with an irregular margin. Neither fine-needle aspiration cytology nor core needle biopsy established a definitive diagnosis. Excisional biopsy was therefore performed. Histologically, the excised tumor tissue results were consistent with a diagnosis of nodular fasciitis of the breast. We report a case of nodular fasciitis of the breast, a rare histological type of breast tumor.

## 1. Introduction

Nodular fasciitis is a benign proliferative lesion that is usually found in the soft tissue of the upper extremity and trunk in young to middle-aged persons. It has rarely been described in the breast. Clinically, the presentation of nodular fasciitis of the breast is similar to that of breast carcinoma. We present a rare case of nodular fasciitis of the breast.

## 2. Case Report

A 35-year-old woman visited a local clinic because of awareness of a mass in her breast. She underwent ultrasonography (US-) guided, fine-needle aspiration biopsy twice. The diagnosis was a “benign” lesion at both times. Four months later she underwent US-guided core needle biopsy. Based on the pathological findings, a phyllodes tumor and a spindle cell carcinoma, among other entities, were included in the differential diagnosis, but there was no definitive diagnosis. She was referred to our hospital for further examination.

Physical examination demonstrated a well-defined, 13 mm hard mass located in the upper inner quadrant of the left breast. No axillary lymph node swelling was found. There was no history of trauma. Her past medical history and family history were negative for malignancy.

**Figure 1:**
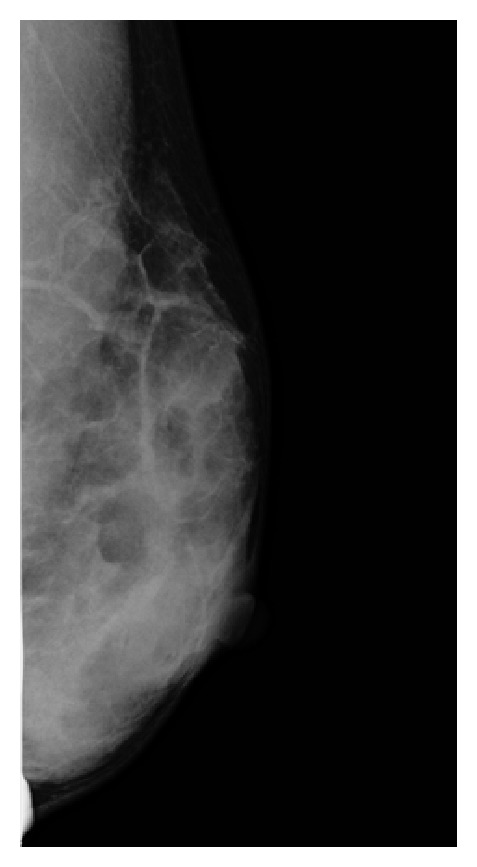
Mammography. The mass was not detected.

**Figure 2:**
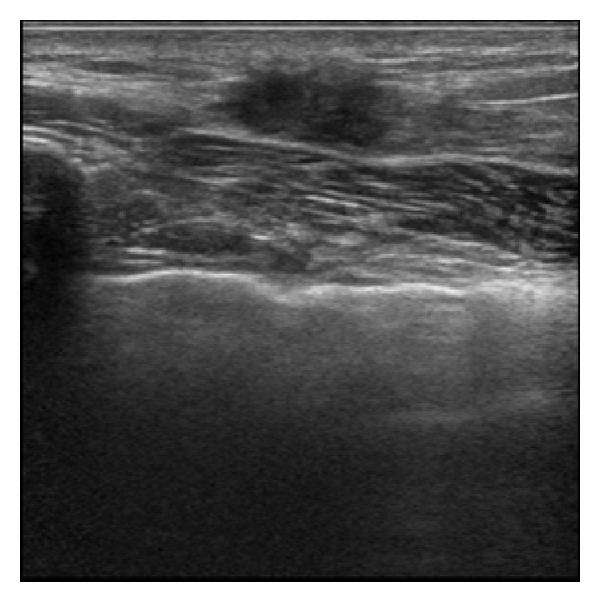
Ultrasonography revealed a hypoechoic lesion with an irregular margin and no acoustic shadow. The mass extended from the anterior border of the mammary gland.

**Figure 3:**
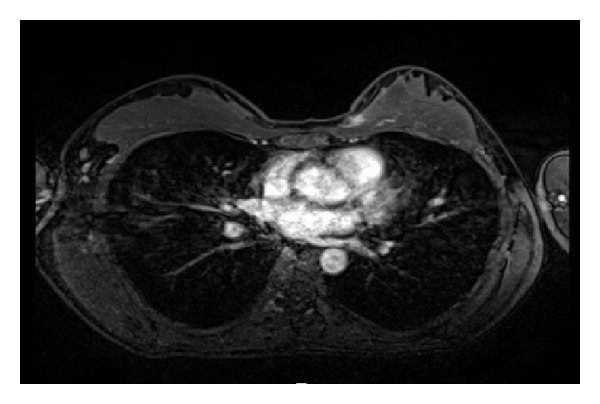
Magnetic resonance imaging (T1-weighted image, fat suppression) showed enhancement of the lesion.

Mammography of the left breast showed a dense breast but no mass (Figure 1). US revealed a hypoechoic lesion with an irregular margin and no acoustic shadow (Figure 2). The mass extended from the anterior border of the mammary gland. Magnetic resonance imaging (MRI)—T1-weighted image, fat suppression—showed enhancement of the lesion (Figure 3). The pathological diagnosis was like the above-mentioned; therefore, core needle biopsy was not performed at our hospital. If based on imaging findings, breast cancer could not be excluded. Excisional biopsy with adequate margins was performed. The tumor was found to be widely adherent to the pectoralis major fascia, so the pectoralis major muscle was partially resected as well.

**Figure 4:**
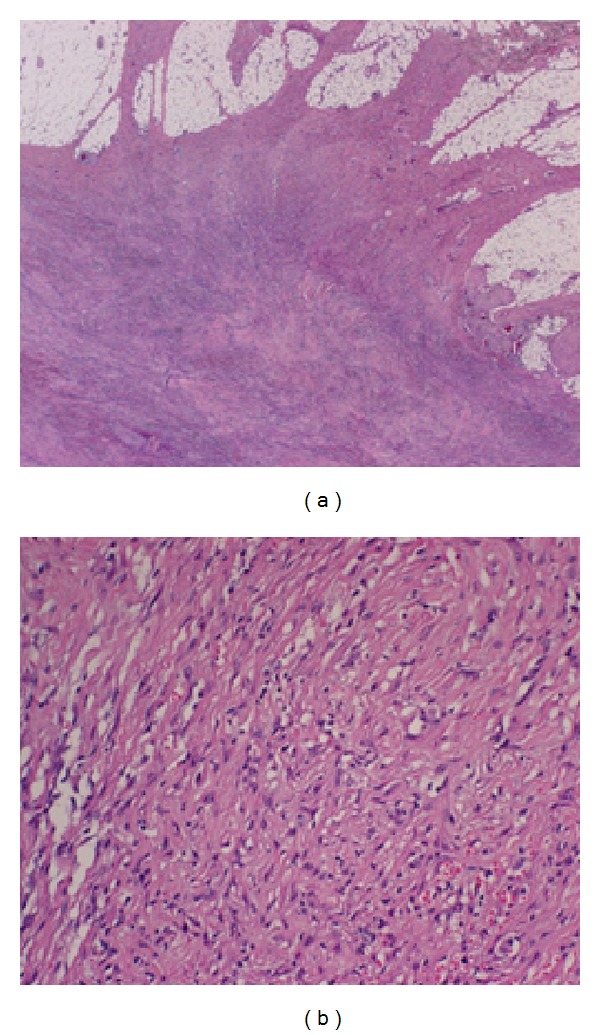
Hematoxylin and eosin staining shows the typical features of nodular fasciitis: (a) ×2 and (b) ×20.

Pathologically, the resected specimen, 45 × 12 mm, contained a whitish firm mass that measured 13 × 6 mm. Microscopically, there was spindle cell proliferation of varying cellularity with an irregular infiltrative margin. Many cellular areas showed the growth of short bundles of spindle-shaped cells in a storiform pattern. The tumor margin was unclear, and the tumor involved the pectoralis major muscle (Figure 4).

Immunohistochemical staining for CD34, desmin, S-100, and keratin was negative. The cells were positive for actin and CD68, indicating a diagnosis of nodular fasciitis.

## 3. Discussion

Nodular fasciitis, which was first described by Konwaler et al., is a benign proliferation of myofibroblasts characterized by their common location in the subcutaneous tissues of the upper extremities and the head and neck. The most characteristic feature of nodular fasciitis is a single, rapidly growing mass. Nodular fasciitis in the breast has been rarely described, with only 21 such reports to our knowledge (Table 1) [1–9, 11–21]. The average age of the patients is 39 years (range 17–84 years). The average size of the lesion is 1.9 cm (range 0.5–5.5 cm). In almost all cases, mammography and US have shown a malignant pattern.

It is believed that local injury may trigger the fibroblastic proliferation [15, 17]. However, the history of trauma was elicited in no more than 10–15% of patients [13]. There was also no history of trauma in our case.

Nodular fasciitis of the breast appears as a hard mass with an irregular shape on both physical examination and radiological evaluation. Because nodular fasciitis has no unique radiological appearance [11], the exact diagnosis can be made only by histopathological examination. Core needle biopsy does not easily diagnose nodular fasciitis because it is difficult to obtain representative cells to make a correct diagnosis [10, 16].

Nodular fasciitis in the breast must be distinguished from benign and malignant breast tumors with nonspecific findings but that are suspected to be malignant [20]. The histological differential diagnosis of nodular fasciitis includes spindle cell tumors such as fibromatosis, myofibroblastoma, spindle cell lipoma, solitary fibrous tumor, phyllodes tumor, spindle cell metaplastic carcinoma, spindle cell melanoma, fibrosarcoma, and leiomyosarcoma. They can be differentiated based on cellularity, nuclear features, collagen content, and growth pattern [16]. Sometimes immunohistochemistry staining (e.g., for CD34) can be helpful with the differential diagnosis [16]. Hence, the diagnosis usually requires histopathological examination of an excisional biopsy [17, 20].

Conservative management or excisional biopsy can be chosen for treatment of nodular fasciitis. A conservative approach is appropriate if the lesion has a typical clinical appearance, with the core biopsy findings consistent with those of nodular fasciitis [13]. In two cases in the literature the lesions were treated successfully with conservative management and disappeared spontaneously. If the criteria for conservative management are not met, however, excisional biopsy is effective, with no further treatment necessary [15, 16]. Recurrence following surgical excision is rare [13, 16, 22].

It is difficult to diagnose nodular fasciitis using imaging or preoperative biopsy. Therefore, to avoid overtreatment, it is necessary to keep the possibility of nodular fasciitis in mind.

## 4. Conclusion

We report a case of nodular fasciitis of the breast, a rare histological type of breast tumor.

## Figures and Tables

**Table 1:** Reported cases of nodular fasciitis in the literature.

Author	Year	Sex	Age	Size (cm)	Mammography	Ultrasonography	Treatmnet
Baba et al. [1]	1978	Female	59	—	—	—	Excised
Fritsches and Muller [2]	1983	—	36	3.0	Unspecified	unspecified	Excised
Torngren et al. [3]	1991	Female	52	1.5	Unspecified	unspecified	Excised
Stanley et al. [4]	1993	—	—	1.5	Unspecified	unspecified	observed
Benson et al. [5]	1994	—	44	1.5	Unspecified	unspecified	Excised
Black et al. [6]	1994	Female	84	3.4	High density mass with microlobulated margin	—	Excised
Green et al. [7]	1997	—	61	2.5	Unspecified	unspecified	Excised
Kontogeorgos et al. [8]	1988	—	—	—	—	—	—
B. Maly and A. Maly [9]	2001	Female	15	2.0	Unspecified	unspecified	Excised
Dahlstrom et al. [10]	2001	Female	38	1.2	High dense mass with indistinct margin	hypoechoic lesion with a smooth margin	Excised
Polat et al. [11]	2002	Female	66	—	High density circumscribed mass	hypoechoic lesion	Excised
Tulbah et al. [12]	2003	Female	18	—	Not performed	Not perfomed	Excised
Brown and Carty [13]	2005	Female	65	5.5	High density mass with microlobulated margin	unspecified	observed
Porter et al. [14]	2006	Female	75	—	High density mass with microlobulated margin	—	—
Porter et al. [14]	2006	Female	52	—	—	hypoechoic round lesion with well-circumscribed margin	—
Hayashi et al. [15]	2007	Female	41	1.5	High density mass with spiculation	hypoechoic lesion with irregular margin	Excised
Squillaci et al. [16]	2007	Male	40	3.5	High density mass with microlobulated margin	hypoechoic lesion with irregular margin	Excised
Ozben et al. [17]	2009	Female	18	0.8	Not performed	hypoechoic lesion	Excised
Iwatani et al. [18]	2012	Female	25	0.5	High density mass with distortion	hypoechoic lesion with irregular margin	Excised
Paker et al. [19]	2013	Male	17	1.5	Not performed	Not perfomed	Excised
Son et al. [20]	2013	Female	41	1.1	High dense mass with indistinct margin	hypoechoic lesion with irregular margin	Excised

F: female; M: male; US: ultrasonography.
